# Molecular detection of *Echinococcus granulosus* and associated risk factors in domestic dogs from a high-altitude endemic region of the Peruvian Andes

**DOI:** 10.14202/vetworld.2026.1149-1162

**Published:** 2026-03-17

**Authors:** Margarita Isabel Huamán Alvites, Oscar Elias Huamán Alvites, William Marcelino Quispe Paredes, Aldo Alim Valderrama Pomé

**Affiliations:** 1 National University of Huancavelica, Huancavelica, Peru; 2 National Institute of Health, Lima, Peru; 3 Faculty of Veterinary Medicine and Animal Science, Micaela Bastidas National University of Apurimac, Abancay, Peru

**Keywords:** *cox1* gene, copro-PCR, cystic echinococcosis, DNA detection, *Echinococcus granulosus*, mitochondrial markers, molecular epidemiology, polymerase chain reaction

## Abstract

**Background and Aim:**

Cystic echinococcosis is a neglected parasitic zoonosis caused by the larval stage of the cestode *Echinococcus granulosus sensu lato*. This study aimed to determine the coprevalence of *E. granulosus sensu lato* in owned domestic dogs and to identify associated epidemiological risk factors in a high-altitude endemic district of the Peruvian Andes.

**Materials and Methods:**

An analytical, community-based, cross-sectional study was conducted in the district of Ascension, Huancavelica, Peru (altitude 3,650 m), from April to December 2021. Simple random sampling selected 159 households across nine communities. Fresh fecal samples were collected from 453 owned dogs. Diagnosis combined conventional coproparasitological examination using the simple flotation technique for helminth eggs and copro-polymerase chain reaction targeting the mitochondrial *cox1* gene for specific detection of *E. granulosus sensu lato* DNA. An interviewer-administered epidemiological questionnaire captured owner demographics, household characteristics, dog management practices, and cohabitation with livestock. Associations were assessed using the Chi-square test, odds ratios, and 95% confidence intervals; statistical significance was defined as p < 0.05. Data were analyzed using SPSS v.25 software (IBM Corp., Armonk, NY, USA).

**Results:**

The overall coprevalence of *E. granulosus sensu lato* was 10.8% (49/453; 95% CI: 7.9–13.8). Village-specific prevalences were highest in Pastales (29.4%) and Sacsalla (16.7%). Canine positivity showed significant associations with household cohabitation with donkeys (p < 0.05) and alpacas (p < 0.01). Households of copro-polymerase chain reaction (PCR)-positive dogs raised significantly more alpacas (mean = 293) than households of negative dogs (mean = 215) (p < 0.01). Concurrent gastrointestinal helminths included *Strongyloides* sp. (7.2%), *Taenia* sp. (6.6%), *Toxascaris* sp. (1.8%), and *Trichuris* sp. (0.4%). Statistically significant coinfection patterns were observed between *E. granulosus sensu lato* and both *Taenia* sp. and Strongyloides sp. (p < 0.01 each). No significant associations emerged with dog sex, owner educational level, housing materials, water source, or sanitation infrastructure.

**Conclusion:**

Active transmission of *E. granulosus sensu lato* is confirmed in domestic dogs in this high-altitude Andean setting, perpetuated primarily by the dog–alpaca epidemiological interface and likely facilitated by unsupervised home slaughter and raw offal feeding practices. The moderate-to-high coprevalence, together with identified livestock cohabitation risk factors and helminth coinfection patterns, indicates a persistent zoonotic threat to human health. Implementation of a One Health strategy, including quarterly praziquantel-based canine deworming, regulation of domestic slaughter, secure offal disposal, and sustained community health education, is urgently needed to interrupt the domestic cycle and reduce the burden of human cystic echinococcosis in the region.

## INTRODUCTION

Cystic echinococcosis is a parasitic zoonosis caused by the larval stage of the cestode *Echinococcus granulosus sensu lato* [1]. This parasite requires two mammalian hosts to complete its life cycle. Domestic dogs and other wild canids act as definitive hosts harboring the adult parasite, whereas herbivorous mammals such as sheep, goats, cattle, pigs, horses, camelids, and cervids serve as intermediate hosts carrying the larval stage [2]. Humans become accidental intermediate hosts when they ingest eggs shed in the feces of infected canids [3].

In South America, cystic echinococcosis occurs in Argentina, Bolivia, Chile, Peru, and Uruguay, as well as in southern Brazil, where domestic dogs constitute the principal definitive hosts [4]. The disease is considered neglected and disproportionately affects populations living in rural and economically disadvantaged areas [5]. Several factors contribute to transmission in these communities, including the close coexistence of dogs with humans and livestock, low socioeconomic status, and inadequate hygienic practices. Risk behaviors include feeding dogs raw viscera from animals slaughtered at home, close interaction with dogs, consumption of untreated food and water, and limited veterinary oversight in dog deworming and livestock slaughter processes [6, 7].

Environmental conditions also influence the persistence of the parasite. Eggs of *E. granulosus* can survive for extended periods under low-temperature conditions, remaining viable in the environment for up to 28 days; however, they survive only a few minutes at high temperatures [8]. After ingestion by intermediate hosts, the parasite develops into hydatid cysts that primarily establish in the livers and lungs of humans and herbivorous livestock. When infected animals are slaughtered and contaminated viscera are improperly disposed of, dogs may ingest these tissues, thereby completing the domestic transmission cycle between dogs and livestock [9].

Control strategies for echinococcosis have included vaccinating livestock and regularly deworming dogs. Since 2009, the EG95 vaccine has been successfully implemented in control programs in Río Negro Province, Argentina, in combination with systematic canine deworming. Similarly, vaccination campaigns in the Aysén region of Chile have significantly reduced the prevalence of hydatid cysts in sheep after 3 years of implementation. Effective control programs must also consider sociocultural factors, particularly in indigenous and rural communities. Evidence indicates that sustained canine deworming and systematic vaccination of sheep and goats represent the most effective strategies for reducing transmission, especially when implemented until all susceptible grazing animals have been replaced [10].

Vaccination of livestock effectively reduces transmission of *Echinococcus* to intermediate hosts; however, vaccine development for definitive hosts, particularly dogs, remains challenging. Several scientific gaps persist regarding protective immune responses in dogs, reproducibility of experimental results, factors influencing immunity, resistance to reinfection, possible age-related decreases in parasite burden, and mechanisms associated with anti-fertility effects. These challenges must be addressed to develop effective canine vaccines [11].

International guidelines also support integrated control measures. The recommendations of the World Health Organization (WHO) and the World Organization for Animal Health (WOAH) are included in the “Roadmap for Neglected Tropical Diseases 2021–2030” and updates to the Terrestrial Animal Health Code. These frameworks emphasize three principal pillars for the control of cystic echinococcosis caused by *E. granulosus*: (a) a One Health approach involving collaboration among human health, animal health, and environmental sectors to interrupt the transmission cycle; (b) animal health interventions such as canine treatment, vaccination of sheep, and improved slaughterhouse control; and (c) human health strategies focused on diagnosis, cyst classification, treatment, and prevention [12, 13].

In Peru, the central and southern highlands provide favorable ecological and epidemiological conditions for the maintenance of the life cycle of *E. granulosus*, and these areas are recognized as endemic zones for human and animal cystic echinococcosis [14]. This situation is exacerbated by the limited infrastructure of slaughterhouses in rural areas [15], many of which operate without authorization from the National Agrarian Health Service of Peru, thereby becoming potential sources of infection. Furthermore, the geographical isolation and long travel distances characteristic of rural Andean regions hinder the transport of livestock to regulated slaughter facilities. Consequently, animals are often slaughtered at home without veterinary supervision [16], which represents one of the principal routes of infection for domestic dogs [17].

This context has important implications for zoonotic transmission. In the Huancavelica region, human cystic echinococcosis seropositivity has been reported at 7.1% [18]. Socioeconomic and environmental factors also play a critical role in disease transmission. Socioeducational characteristics, such as the age and educational level of dog owners, influence their capacity to understand and apply preventive measures. Basic sanitation conditions, including water sources and toilet availability, determine the risk of fecal–oral contamination. Structural housing characteristics, particularly flooring materials, may also influence parasite survival because dirt floors retain moisture and are more difficult to disinfect, thereby facilitating egg persistence in the environment.

The district of Ascension is predominantly rural and characterized by the extensive rearing of alpacas, llamas, and sheep [19]. In this area, livestock farmers often have limited formal education and live in precarious housing conditions. Home slaughter of livestock is common, and dogs are frequently fed raw viscera. These practices increase the risk of maintaining the parasite’s transmission cycle. Therefore, diagnosing infection with *E. granulosus* in owned domestic dogs is essential to understanding the epidemiology of this parasitosis and its implications for public health [16].

Consequently, the objective of the present study was to determine the coprevalence of *E. granulosus* in domestic dogs in the district of Ascension, Huancavelica, Peru.

## MATERIALS AND METHODS

### Ethical approval

The study protocol included an interview guide and an informed consent form for dog owners. The Research Ethics Committee of the National University of Huancavelica approved this protocol (Resolution No. 0791-2020-CU-UNH dated December 14, 2020). Dog owners signed the informed consent form, agreeing to participate in the survey, and authorizing the collection of fecal samples from their dogs. Owners’ participation was completely voluntary; therefore, there was no coercion or undue incentives to participate, and they understood the objectives, procedures, and potential risks or benefits of the study. Participation was anonymous to maintain community confidentiality when identifying disease outbreaks.

The protocol complied with the institutional animal welfare guidelines required by Peruvian regulations regarding the non-invasive handling of farm animals. The Peruvian Law on Animal Protection and Welfare (Law No. 30407) established a duty to ensure animal welfare and to avoid unnecessary suffering during any procedure, including sample collection. International guidelines from the WOAH (Terrestrial Code) were also followed as a basis for animal management and welfare, including pain and stress management and proper dog restraint to minimize discomfort. Furthermore, a refinement was implemented, as trained personnel performed rectal swab testing using techniques that minimized pain and stress. Finally, the Animal Research: Reporting of *In Vivo* Experiments 2.0 guidelines were considered (staff training and experience in sample collection, appropriate restraint and handling methods used with dogs, and appropriate procedures for fecal sample collection).

### Study period and location

**Figure 1:**
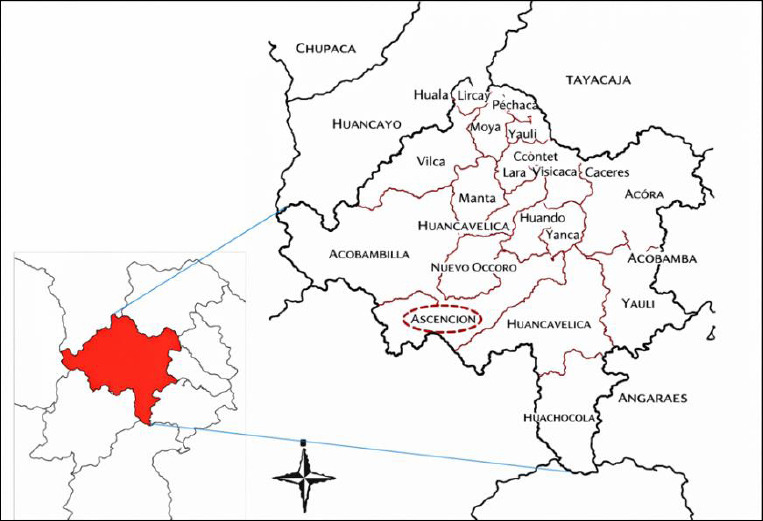
Map of Ascension District, Huancavelica Province, Peru

The study was conducted between April and December 2021. The district of Ascension has an area of 432.2 km² and is located in the province and region of Huancavelica, at an altitude of 3,650 m, at the coordinates: 12°47′02″S and 74°58′42″W [19] (Figure 1). The climate of Ascension is cold, with variations from semi-arid to humid depending on altitude, maintaining an average annual temperature of between 9°C and 11°C. It is characterized by a marked daily temperature range, with days of intense solar radiation and nights with sharp temperature drops. The annual cycle comprises two periods: the rainy season (December–April), with annual precipitation exceeding 800 mm [20], and the dry season (May–November), which is characterized by the absence of rain, clear skies, and the presence of intense frosts at night [21].

Vegetation is closely related to altitude and seasonal rainfall. During the rainy season, abundant herbaceous vegetation predominates, providing livestock with fodder. Bofedales (high Andean wetlands) serve as moisture and quality pasture reserves during drought in higher-altitude areas, along with xerophytic species, low shrubs, and grasses that are resistant to water and heat stress [22].

Recognized as the “Livestock Capital of the Andes,” the economy of Ascension is based on livestock farming. The predominant production system is semi-extensive, in which cattle graze freely on communal or private land with limited supplementation. Production efficiency is classified as low to medium, conditioned by traditional management and the scarcity of nutritious pastures during the dry season [23]. The main species identified are cattle (mainly Brown Swiss and Creole crosses, with a focus on dairy or dual-purpose production), sheep (for wool and meat production), and South American camelids (alpacas and llamas, strategically located in higher-altitude areas) [24].

### Study design

The study design was non-experimental and analytical because interdependence was measured without manipulating the variables. Furthermore, this was a community-based cross-sectional study conducted between April and December 2021.

### Study population and sample size

A simple random sample was taken from this population using the following formula: n = NZ²PQ / E² (N−1) + Z²PQ. The sample size was calculated based on a normal distribution, which required a sample of 270 households (N), with an estimated proportion of 50% (P), an accepted error of 5% (E), and a confidence level of 95% (Z). A sample of 159 households was estimated, considering that in the rural sector of the Peruvian highlands, there would be 0.6 dogs per household; thus, 95.4 dogs should have been sampled. However, fresh fecal matter from 453 dogs was collected in sterile containers [25], which were inactivated at −70°C for 72 h and stored at −20°C until processing.

Feces samples were collected from three to four dogs per household. The maximum number of dogs sampled per household was four. Only dogs of all ages with owners who permanently resided in the Ascension district were sampled. We excluded dogs with clinical signs or symptoms (apparently ill), stray dogs, puppies younger than 3 months, or dogs that had been dewormed with praziquantel in the last 30 days were excluded.

The inhabitants of the Ascension District have one of the lowest average incomes in the country and a high incidence of poverty, with an average monthly income of $359.2 for dependent household activities and $75.1 for independent activities. The poverty level in this population fluctuates between 32.9% and 36.2%, with extreme poverty between 6% and 7.7%. The main economic sector by employed population is agriculture (73.7%), followed by transportation (8.9%) and other services (17.3%). In addition, the illiteracy rate in the population aged 15 and over is 17.7%, which is significantly higher than the national average, with a notable gap between urban areas (9.2%) and rural areas (21.5%), as well as between men (8.4%) and women (26.2%). Conversely, the educational level of the population aged 15 and over is as follows: secondary education, 37%; primary education, 27.7%; higher education (non-university and university), 19.0%; and no educational level, 16% [19].

### Data collection

An epidemiological survey was conducted through an interview with heads of household to evaluate the factors associated with echinococcosis in dogs. The survey recorded personal data of the owners of the dogs (age, sex, level of education, place of residence, and characteristics of their home) and characteristics of the dogs (age, sex, coexistence with other animals, and coinfection with gastroenteric parasites), as well as risk practices related to animal breeding and visceral disposal. This study was conducted in coordination with the Regional Health Directorate and the District Municipality of Ascension. The questionnaire underwent initial pilot testing with 10% of the calculated sample in a community with characteristics similar to Ascension. Expert judgment was used to validate the content to ensure that the language was culturally appropriate for the area. The mode of administration was face-to-face (in-person) interviews conducted at the home of the participants. Each interview lasted 15–20 min. The responses were recorded on physical forms (paper) and then digitized into an electronic database (Excel, Office 365, Microsoft Corp., Washington, USA).

### Collection and handling of fecal samples

Sampling was conducted during the dry season, between April and November 2021, when camelid management is more active and often coincides with festivities or slaughter periods, during which dogs have greater access to raw offal. The feces were collected by a team of veterinarians and biologists who had been trained not only to administer the survey but also to handle dog fecal samples. The samples were obtained directly from the dogs’ rectum and from the superficial part of fresh feces found in the house, which were preserved in a disposable container and then labeled and transported, maintaining the cold chain, to the National Reference Laboratory of Parasitic Zoonosis of the National Center for Public Health (CNSP) of the National Institute of Health (INS), in the city of Lima, Peru. Fecal samples were collected from dogs of all ages in their respective homes. Dogs that showed signs or symptoms (apparently ill) were excluded.

To avoid direct contact between the handler and the feces, disposable, waterproof, and resistant gloves (nitrile or latex), a face mask, a disposable apron or gown, and safety goggles were required. It was also necessary to wash hands with soap and water, followed by an alcohol-based hand sanitizer, before and after removing personal protective equipment.

Rectal swab testing was used to ensure the sample belonged to the dog and was as fresh as possible to prevent DNA degradation. However, sampling of fresh feces from the ground was performed only when rectal sampling was not possible due to the animal’s aggressive temperament or if the dog had just defecated and the rectum was empty. Thermal management of the samples was critical to preserving the integrity of the eggs and genetic material. The samples were immediately placed in thermos flasks containing cooling gels (4°C–8°C). The maximum time between home collection and arrival at the local freezing center did not exceed 6 h. The samples were initially stored at −20°C for 48–72 h while the transport batch was completed. However, the samples were transported to the laboratory in validated thermal boxes. Upon arrival at the laboratory, the samples were transferred to −70°C ultra-freezers for long-term storage prior to polymerase chain reaction (PCR) analysis. Each sample was placed in sterile 60 mL polypropylene screw-cap bottles. Each bottle was labeled with indelible ink and a transparent tape-protected sticker that included the dog’s unique code, housing code, exact date and time of collection, and district sector.

Given the high infectivity of *E. granulosus* eggs to humans, disposable nitrile gloves (changed between each dog), N95 masks (to prevent inhalation of dry fecal dust in the yards), waterproof aprons, and eye protection were used during rectal manipulation. After sampling, 70% alcohol and hypochlorite solutions were used to disinfect the support materials and the external surfaces of the jars.

The study design focused exclusively on dogs with owners, collecting samples in home yards and interviewing the “heads of household.” Therefore, stray dogs (street dogs or vagrants) were not sampled because their existence in the area is unknown and there is no municipal slaughterhouse in the district where dogs could have access to raw offal.

To avoid cross-contamination of samples, trained professionals (veterinarians and biologists) collected the samples directly from the dog’s rectum. Therefore, the use of disposable containers and mass transport requires very strict handling to avoid contact between samples.

To reduce the PCR inhibition rate in fecal samples, stool-specific extraction kits and sample dilution protocols were used.

### Coproparasitological examination

For the coproparasitological diagnosis, the simple flotation technique was used, sifting the feces and taking 2–3 g to be suspended in a 0.85% saline solution, which was filtered in a 0.12 μm metal strainer; then, 12 mL of sample was added to the 15 mL conical tubes to be centrifuged at 1,800 rpm for 5 min. The supernatant was removed, and 0.85% saline solution was added, then the mixture was centrifuged again. This process was repeated until the supernatant was translucent. The samples were observed under a microscope, and the identification and quantification of eggs per gram of feces were performed based on the general characteristics described by Hendrix [26].

The simple flotation technique is a standard method for screening eggs of the Taeniidae family, with an estimated sensitivity of 60%-80% in mild infections due to intermittent egg elimination in the feces. In terms of specificity, it is highly effective in identifying the genus. To ensure the reliability of coproparasitological findings, the following measures were implemented: A second expert analyst independently re-examined 10% of samples (n = 45); Cohen’s kappa index was calculated, obtaining a value of 0.82, which represents a “near-perfect” level of agreement according to the Landis and Koch scale (interobserver reliability); a blind reading was performed, in which analysts conducted the microscopic examination without prior knowledge of the sample’s origin or the results of cohabitation with livestock, thus eliminating possible observation biases; and duplicates of samples that initially tested negative but came from areas with high livestock density were analyzed to minimize the false negative rate.

A 0.85% saline solution was used exclusively for the initial homogenization of the sample and for sieving macroscopic debris during the washing and homogenization phases. A saturated solution of sodium nitrate (NaNO_3_) or Sheather’s solution (saturated sugar) with a specific gravity of 1.20–1.27 was used for the recovery of *Echinococcus*/Taenia eggs. This ensures that the eggs, which have a lower density, float to the surface while the heavy debris settles. Observation was performed using a binocular optical microscope with an initial scanning magnification of 10× (to locate suspicious structures) and morphological confirmation at 40× (to identify internal hooks and the striated membrane characteristic of eggs of the Taeniidae family). Finally, a double-blind validation was performed to ensure inter-observer agreement, with each slide being independently read by two trained biologists. In case of discrepancy in the result (positive/negative), a third review was performed by a supervisor from the INS National Reference Laboratory.

### Copro-PCR for *E. granulosus*

For copro-PCR diagnosis, the sifted stool samples were thawed, and genomic DNA was extracted using the commercial SIGMA kit (Sigma-Aldrich, USA) following the manufacturer’s procedure, with minor modifications to concentrate the genomic DNA from the samples. A pair of primers was used to amplify *E. granulosus* DNA, selected from the mitochondrial gene *cox1* (GenBank accession no. AF297617); primer sequences CO1-F (5′-TTTTTTGGCCATCCTGAGGTTTAT-3′) and CO1-R (5′-TAACGACATAACATAATGAAAATG-3′), producing an amplicon of 460 bp. These primers amplify DNA from almost all *E. granulosus* strains; therefore, the primer pair was selected for the identification of the *cox1* gene segment, which is intended to serve as an amplification target for the copro-PCR diagnosis of *E. granulosus* spp. in dog feces [27].

The PCR reaction was performed in a final volume of 25 μL, containing 25 μL DreamTaq 2× Green PCR Master Mix (Thermo Scientific, Waltham, MA, USA), 18.5 μL nuclease-free water, 0.5 μL each primer, and 2 μL genomic DNA. PCR amplification was performed on the Mastercycler Pro S & Control Panel (Eppendorf, Hamburg, Germany) using the following thermal conditions: first, an initial denaturation cycle at 94°C for 4 min. Then, 38 denaturation cycles of 94°C for 30 s, 55°C for 30 s, and 72°C for 30 s were performed. Third, the final elongation was performed at 72°C for 5 min, and the sample was stored at 4°C in the same instrument. The amplified products were confirmed and analyzed using 1.5% agarose gel electrophoresis. Electrophoresis was performed for 45 min, with 80 V for the first 15 min and 120 V for the next 30 min [1, 6].

To ensure the validity of the molecular diagnosis of *E. granulosus*, critical performance controls (positive and negative) were implemented: Positive Control, where genomic DNA from *E. granulosus* (locally confirmed strain) was included in each PCR run to validate the efficiency of amplification and the integrity of the reagents (master mix); negative control (NTC), where a contamination-free control (molecular biology grade water) was used in each plate/set of tubes to detect any traces of contamination during reaction preparation; and extraction control, where a known negative stool sample was processed during the DNA extraction phase to monitor the cleanliness of the kits and lysis reagents.

Area segregation was considered in terms of contamination prevention measures, where the workflow was carried out in separate and physically isolated rooms for the three critical stages: (a) preparation of the PCR master mix, (b) DNA extraction from fecal samples, and (c) amplification/analysis of products (post-PCR). Physical barriers were also used, with pipetting and reaction preparation carried out in a laminar flow hood under ultraviolet (UV) light for decontamination before and after use. Only filter tips (aerosol-resistant tips) were used.

Finally, all samples were processed in duplicate. In cases of discrepancy between the duplicates (one positive and one negative), a third confirmation run was performed. Only samples with consistent amplification in both tests were considered “positive.”

The presence or absence of *Echinococcus* spp. nucleic acids was determined by quantifying only the number of positive dogs to determine the prevalence of this parasitic disease; however, circulating genotypes were not identified.

### Statistical analysis

Data analysis and processing were performed using SPSS v.25 (IBM Corp., Armonk, NY, USA). The categorical variables were statistically contrasted using the Chi-square independence test, with the asymptotic or exact method, and the odds ratio with 95% confidence intervals. A p-value ≤ 0.05 was used to indicate statistical significance.

Univariate and multivariate logistic regression analyses were performed to assess potential associations. Although variables with significant associations were identified for canine echinococcosis, multivariate analysis was not carried out due to collinearity among some factors evaluated. Missing data were handled using the Maximum Likelihood Models technique to avoid bias and loss of statistical power, using all available information to estimate the model parameters without the need for explicit imputation, assuming that the missing data were random.

The Hosmer–Lemeshow test was used to assess model goodness of fit and evaluate whether there was a significant difference between the observed and expected frequencies of positive cases of echinococcosis across the model-defined risk groups. Confounding factors, referring to external variables associated with both exposure and outcome, were controlled for by performing a multivariate logistic regression in which the confounding factors (owner’s gender and educational level, dog’s area of residence, type and materials of housing, water supply, type of drainage, open defecation, night light generator, coexistence with other animals, number of animals raised, and parasitic coinfection) were included as independent variables in the model along with the main variable of interest. The model calculated the adjusted odds ratio (OR), which represents the association of interest after “neutralizing” the effects of confounding variables.

For prevalence and biological factors, the unit of analysis was the dog, but for environmental and management factors (such as offal disposal), the unit was the household. Listwise deletion was used to handle missing data; that is, only subjects with complete information for all variables analyzed were included in the final models. An exploratory analysis was performed using Pearson’s Chi-square test to identify individual associations with *E. granulosus* positivity. Variables were coded to facilitate the interpretation of the OR: binary (dichotomous): presence of the condition = 1, absence = 0. Dummy variables were used for categorical/ordinal variables to establish a reference category.

**Figure 2:**
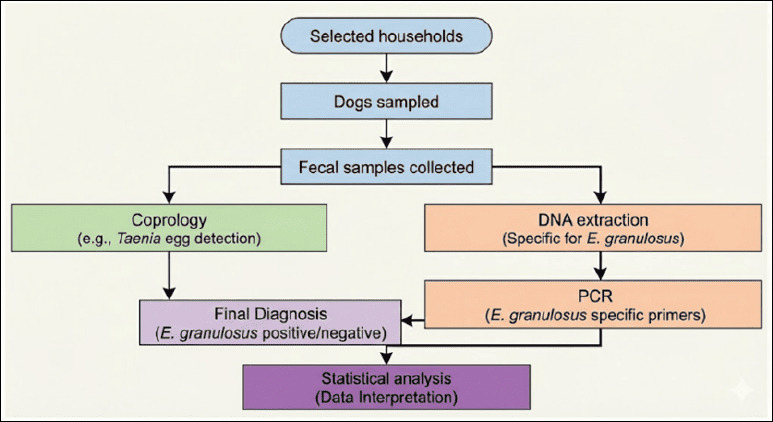
Methodological workflow for *Echinococcus granulosus* detection

A multivariate model (logistic regression) was used to adjust for possible confounding variables and identify independent predictors of infection. All variables with a p-value ≤ 0.20 in the univariate analysis were included. This preventive threshold ensured that variables, although not significant on their own, contributed to the model in the presence of other variables were not discarded. The backward stepwise method based on likelihood was used. A multicollinearity test was performed to assess the scientific validity of the inferences, with variance inflation factors (VIFs) calculated for the predictors. A VIF >5 was considered indicative of multicollinearity, and redundant variables were eliminated or combined. In addition, the Hosmer–Lemeshow test was applied, with a p-value >0.05 indicating that the model adequately fit the observed data. Figure 2 shows the methodological workflow for detecting *E. granulosus* in dogs.

## RESULTS

### Owner and dog characteristics

Most dog owners were male (61.1%) and had a primary education (58.5%) (Table 1). The average age of the dogs was 3.9 (SD = 3.5) years and that of their owners was 53.9 (SD = 17) years. 68.5% (371/542) of the dogs were male and 31.5% (171/542) were female. All the dogs were mixed breeds, used for guarding and herding. The study showed that 47.3% of dogs were fed offal containing cysts at some point, and 7% were fed cooked offal. None of the dogs were confined because homes in rural areas do not usually have a complete perimeter fence, allowing dogs to move freely inside and outside the home. It was found that 65.4% of dogs had been dewormed at some point, and 89.6% had been dewormed within the last year.

**Table 1:** Dog owners’ general data.

Variable	n	%
Sex (n = 108)		
Female	42	38.9
Male	66	61.1
Level of education (n = 106)		
No studies	3	2.8
Primary	62	58.5
High school	37	34.9
Superior	4	3.8

### Prevalence of canine echinococcosis by village

On the other hand, as shown in Table 2, the prevalence of *E. granulosus* in dogs was 10.8% (49/453; 95% CI = 7.9–13.8). In addition, the villages with the highest prevalence of echinococcosis were Pastales and Sacsalla, with 29.4% (95% CI = 17.9–40.9) and 16.7% (95% CI = 6.4–26.9), respectively. The other villages had a prevalence of 8%. Sex was not associated with echinococcosis (p > 0.05).

**Table 2:** Prevalence of canine echinococcosis according to the village of origin in Ascension, Huancavelica.

Village	Positive (n)	%	Negative (n)	%	Total (n)	p-value
Cachimayo	3	2.9	99	97.1	102	0.000
Cotay	3	7.9	35	92.1	38	
Cusibamba	3	3.3	87	96.7	90	
Lachocc	2	5.3	36	94.7	38	
Pastales	20	29.4	48	70.6	68	
Sacsalla	10	16.7	50	83.3	60	
Telapaccha	1	4.3	22	95.7	23	
Doctoral	7	5.6	117	94.4	124	
Total	49	10.8	404	89.2	453	

Percentages correspond to the proportion of positive or negative dogs within each village. The p-value corresponds to the Chi-square test evaluating the association between village of origin and *Echinococcus granulosus* infection in dogs.

### Housing characteristics and echinococcosis

Table 3 shows that none of the characteristics of the dwelling (housing material, type of dwelling, water supply, toilets, and energy source) were associated with echinococcosis in dogs (p > 0.05).

**Table 3:** Association between canine echinococcosis and housing characteristics of dog owners in Ascension District, Huancavelica.

Characteristics	Positive (n)	%	Negative (n)	%	Total (n)	p-value
Housing material						0.091
Adobe/rammed earth	19	7.0	254	93.0	273	
Stone	30	11.1	240	88.9	270	
Type of housing						0.831
Family	16	9.4	154	90.6	170	
Multifamily	33	8.8	340	91.2	373	
Water supply						0.278
Public network	0	0.0	16	100.0	16	
Subsurface well	0	0.0	10	100.0	10	
River/ditch	10	7.2	128	92.8	138	
Spring	39	10.3	340	89.7	379	
Toilets						0.281
Silo	12	6.9	161	93.1	173	
Homemade latrine	9	7.6	109	92.4	118	
It does not have	28	11.1	224	88.9	252	
Defecate in the open air	28	10.8	232	89.2	260	0.174
Power source						0.057
Electrical fluid	0	0.0	46	100.0	46	
Solar panel	43	9.5	409	90.5	452	
Candle	6	13.3	39	86.7	45	

The p-values were obtained using the Chi-square test to evaluate the association between housing characteristics and *Echinococcus granulosus* infection in dogs. No statistically significant association was observed (p > 0.05).

### Coexistence with other animals

The coexistence of dogs with donkeys (p < 0.05) and alpacas (p < 0.01) in the same household was associated with echinococcosis in dogs (Table 4).

**Table 4:** Association between canine echinococcosis and livestock species raised in the same household in Ascension District, Huancavelica.

Species	Positive (n)	%	Negative (n)	%	Total (n)	p-value
Ovine	48	9.5	456	90.5	504	0.144
Goats	0	0.0	13	100.0	13	0.250
Pigs	0	0.0	29	100.0	29	0.081
Bovines	16	8.2	178	91.8	194	0.638
Equines	10	10.9	82	89.1	92	0.498
Donkeys	4	40.0	6	60.0	10	0.001
Alpacas	48	10.0	430	90.0	478	0.025
Llamas	21	7.9	245	92.1	266	0.368

The p-values were obtained using the Chi-square test to evaluate the association between livestock species raised in the household and *Echinococcus granulosus* infection in dogs. Statistically significant associations were observed for donkeys (p = 0.001) and alpacas (p = 0.025).

### Gastrointestinal parasites and coinfections

The stool examination showed that the dogs were infected with different parasites, such as *Strongyloides* sp. (7.2%; 39/536; 95% CI = 4.9–9.5), *Taenia* sp. (6.6%; 36/536; 95% CI = 4.5–8.8), *Toxascaris* sp. (1.8%; 10/536; 95% CI = 0.6–3.1) and *Trichuris* sp. (0.4%; 2/536; 95% CI = 0.1–1.3). Table 5 shows that the coinfection of *E*. *granulosus* with Taenia sp. and *Strongyloides* sp. showed a statistically significant association (p < 0.01). However, coinfections with *Trichuris* sp. and *Toxascaris* sp. were not associated (p > 0.05).

**Table 5:** Association between canine echinococcosis and co-infecting intestinal parasites in dogs from Ascension District, Huancavelica.

Co-infecting parasites	Positive (n)	%	Negative (n)	%	Total (n)	p-value
*Taenia* sp.	9	25.0	27	75.0	36	0.001
*Strongyloides* sp.	15	38.5	24	61.5	39	0.000
*Trichuris* sp.	0	0.0	2	100.0	2	0.655
*Toxascaris* sp.	1	10.0	9	90.0	10	0.913

The p-values were obtained using the Chi-square test to assess the association between *Echinococcus granulosus* infection and coinfection with other intestinal parasites. Statistically significant associations were observed for *Taenia* sp. and *Strongyloides* sp. (p < 0.01).

### Number of animals raised and echinococcosis

No statistically significant difference was found between the age of the owners and echinococcosis in dogs (p > 0.05), nor was there a difference between the number of inhabitants in the house and echinococcosis in dogs (p > 0.05). Table 6 shows the difference between the number of alpacas raised and the positivity of echinococcosis in dogs (p < 0.01). So much so that in the homes of dogs with echinococcosis, more alpacas were raised (x̅ = 293) compared with the homes of dogs that tested negative (x̅ = 215). The number of other bred animals showed no statistically significant difference with echinococcosis in dogs (p > 0.05).

**Table 6:** Association between *Echinococcus granulosus* infection in dogs and the number of livestock species raised in households in Ascension District, Huancavelica.

Species	p-value	Mean difference	Standard error difference	95% CI
Sheep	0.175	−17.775	13.019	−43.554 – 8.004
Dogs	0.307	−0.233	0.227	−0.683 – 0.217
Cows	0.199	6.955	5.333	−3.786 – 17.695
Alpacas	0.006	−78.014	27.817	−133.125 – −22.903
Llamas	0.864	−2.300	13.416	−29.118 – 24.518
Horses	0.248	0.850	0.714	−0.640 – 2.340

The p-values correspond to statistical comparisons between households with *Echinococcus granulosus*-positive dogs and those with negative dogs. A statistically significant difference was observed only for alpacas (p = 0.006), indicating that households with infected dogs tended to raise a higher number of alpacas. CI = Confidence interval.

## DISCUSSION

### Prevalence of canine echinococcosis in Ascension

The prevalence of *Echinococcus granulosus* in dogs in this study (10.8%) was similar to that found in Uganda [28], India [29], Iran [30, 31], Turkey [32], Kenya [33], and Israel [34]; it was only higher than that reported in the Falkland Islands [35] but lower than that reported in Iraq [36], Iran [37], and Morocco [38]; therefore, Ascension is recognized as a rural, remote, and epidemiologically underserved area. Demonstrating active parasite circulation fills an important knowledge gap in national surveillance.

### Socio-demographic characteristics of dog owners

The study showed that the average age of dog owners was 54 years, and half had only primary education (50.6%), unlike another study conducted in Andahuaylas, Peru, where most dog owners had higher education [39]. Consequently, having a primary education would imply that knowledge about dog ownership is precarious and that there is a greater risk of becoming infected with CEC [11].

### Influence of altitude and geographic location

Dogs in some villages had a higher prevalence of echinococcosis (Pastales and Sacsalla), because the localities in the Suni altitudinal zone (3500–4000 m.a.s.l.) have a higher prevalence than those in the Quechua zone (2300–3500 m.a.s.l.) [2]. Although altitude is not a direct factor in causing this disease, it does affect prevalence, particularly in mountainous areas where interactions between domestic and wild animals are common [40].

### Association with the sex of dogs

Keyhani *et al*. [20] found no association between dog sex and echinococcosis. However, in other research [41, 42], it is mentioned that sex is a risk factor for contracting *E. granulosus*, especially for male dogs, because people prefer to have male dogs as they better protect the home and livestock and because they do not reproduce unlike females [42].

### Housing conditions and echinococcosis risk

House characteristics, such as housing material and type, water supply, toilets, and energy source, were not associated with echinococcosis in dogs. This result is consistent with reports from other Peruvian regions, likely because this parasitosis is endemic throughout the district [2].

### Role of alpaca husbandry in transmission

The coexistence of dogs and alpacas in the same house was associated with echinococcosis in dogs, probably because dogs accompany livestock during grazing [41], resulting in continuous contact that increases the risk of disease [43]. However, the genotypes G7 (*E. canadensis*) [10] and G1 (*E. granulosus* sensu stricto) have been reported in alpacas from endemic regions of Peru, which is the most common strain identified in humans and animals in endemic regions around the world [44].

### Coexistence with donkeys

An association was also found between the coexistence of dogs and donkeys; however, this association may be due to chance, given the small number of donkeys raised in the Ascension houses. However, another study found that sheepdogs that lived with donkeys were less likely to be infected with echinococcosis, because people who raise donkeys tend to have higher incomes than those who do not. Consequently, dogs from higher-income households may be better fed and less likely to snoop around in the garbage [45].

### Gastrointestinal parasite coinfections

Stool examination showed that the dogs were infected with *Strongyloides* sp., *Taenia* sp., *Toxascaris* sp., and *Trichuris* sp., as reported in previous studies [33, 34, 46, 47]. However, a statistically significant association was found between the coinfection of *E. granulosus* with *Taenia* sp. and *Strongyloides* sp. Coinfection with *Taenia* sp. was also reported by Hartnack *et al*. [47], who noted that dogs can be infected with both *Taenia* sp. and *Echinococcus* spp. through predation and local prey species are infected with metacestodes of both genera. However, no association between *E. granulosus* and other parasites has been reported [34]. The findings show a strong statistical association between *E. granulosus* infection and coinfection with *Strongyloides* sp. and *Taenia* sp. (p < 0.01). Few studies in Latin America have explored coinfections in detail. This adds originality by highlighting the ecological overlap between helminths in dogs. Coinfection profiles could serve as predictive markers of *Echinococcus* risk.

### Livestock herd size and infection risk

The study also showed that in the homes of dogs with echinococcosis, more alpacas were bred (x̅ = 293) compared to the homes of dogs that tested negative, in whose homes the number of alpacas bred was lower (x̅ = 215), which shows that the larger size of the alpaca herds would constitute a risk factor [28], since the breeding of these animals is inadequate in Huancavelica because it is done only as a source of food in pastures of low bearability and with deficient sanitary care [48]. A critical finding is the direct association between the number of raised alpacas and positivity in dogs. Unlike reports from other high Andean areas where sheep are the main reservoir, alpacas have emerged as a key epidemiological node in Ascension. The fact that households with infected dogs have larger herds suggests that, as production scale increases, the likelihood of home slaughter and accidental access by dogs to infected viscera also increases, reinforcing the importance of this camelid in the human transmission chain.

## CONCLUSION

This study demonstrated an active transmission cycle of cystic echinococcosis in domestic dogs in the district of Ascension, Huancavelica, Peru. The prevalence of *E. granulosus* in dogs was 10.8% (49/453; 95% CI = 7.9–13.8), confirming the persistence of this zoonotic parasite in a rural Andean ecosystem. Higher prevalence was observed in specific villages, particularly Pastales and Sacsalla, suggesting localized transmission foci. In addition, the coexistence of dogs with alpacas (p < 0.01) and donkeys (p < 0.05) was associated with infection, and households with infected dogs maintained significantly larger alpaca herds compared with households with non-infected dogs. Coproparasitological findings also revealed that dogs were commonly infected with other gastrointestinal helminths, including *Strongyloides* sp., *Taenia* sp., *Toxascaris* sp., and *Trichuris* sp., with statistically significant associations between *E. granulosus* infection and coinfection with *Strongyloides* sp. and *Taenia* sp. (p < 0.01). These results indicate that ecological and livestock management factors contribute substantially to parasite transmission in this high Andean environment.

From a practical perspective, the findings highlight several critical public health implications. The widespread practice of home slaughter and improper disposal of infected viscera likely facilitates access of dogs to parasite stages, thereby sustaining the domestic transmission cycle. The association between large alpaca herds and infection risk suggests that camelid husbandry practices play a key epidemiological role in this region. Therefore, integrated control strategies should include regular deworming of dogs, improved management and disposal of livestock viscera, health education programs targeting rural communities, and coordinated surveillance within a One Health framework that integrates veterinary and public health services.

A major strength of this study is the combination of coproparasitological examination and molecular detection to confirm *E. granulosus* infection in dogs, which improves diagnostic reliability compared with microscopy alone. Furthermore, the study provides one of the first epidemiological assessments of canine echinococcosis in the Ascension district, thereby filling an important gap in national surveillance data. The relatively large sample size of dogs and the inclusion of socio-environmental variables also strengthen the analytical capacity of the study.

However, several limitations should be considered when interpreting the results. First, the cross-sectional design does not allow the establishment of causal relationships between risk factors and infection. Second, stray dogs were not included in the sampling, which may underestimate the overall prevalence of the parasite in the region. Third, although molecular detection confirmed infection, genotyping of *E. granulosus* strains was not performed; therefore, the circulating genotypes and their relative epidemiological importance remain unclear.

Future studies should focus on molecular characterization of circulating *Echinococcus* genotypes in both intermediate and definitive hosts to better understand transmission dynamics in Andean ecosystems. Longitudinal studies assessing seasonal variation in infection and the effectiveness of dog deworming programs would also be valuable. In addition, investigations integrating livestock infection rates, human seroprevalence, and environmental contamination could provide a more comprehensive understanding of the parasite’s epidemiology within a One Health framework.

In conclusion, the present study confirms the continued circulation of *E. granulosus* among domestic dogs in Ascension and identifies livestock management practices, particularly alpaca husbandry, as important epidemiological factors. Strengthening control programs that combine community education, systematic dog deworming, and improved slaughter and waste management practices will be essential to interrupt the parasite’s transmission cycle and reduce the public health burden of cystic echinococcosis in rural Andean regions.

## DATA AVAILABILITY

All the generated data are included in the manuscript.

## AUTHORS’ CONTRIBUTIONS

MH: Conceived, designed, and coordinated the study and data collection tools. MH, OH, and WQ: Oversaw field sampling, data collection, laboratory work, and data entry. MH and AV: Project management, statistical analysis and interpretation, manuscript writing, and visualization. All authors have read and approved the final version of the manuscript. MH, OH, WQ, and AV: Critical review of the manuscript and compliance with ethical protocols. All authors have read and approved the final version of the manuscript.
